# Integrating Metabolomic and Proteomic Profiles Reveals the Mechanism of Dietary Energy Levels Regulating Milk Performance and Antioxidative Capabilities of Lactating Donkeys

**DOI:** 10.3390/antiox15050528

**Published:** 2026-04-22

**Authors:** Yanli Zhao, Yuanxi Yue, Zhiyi Zhao, Yao Chen, Sumei Yan, Binlin Shi, Zaccheaus Pazamilala Akonyani

**Affiliations:** Key Laboratory of Animal Nutrition and Feed Science of Inner Mongolia Autonomous Region, College of Animal Science, Inner Mongolia Agricultural University, Hohhot 010018, China; ylzhao2010@163.com (Y.Z.); yueyuanxi2013@163.com (Y.Y.); zyzhao1206@163.com (Z.Z.); 19997636168@163.com (Y.C.); shibinlin@yeah.net (B.S.); zakonyani@hotmail.com (Z.P.A.)

**Keywords:** antioxidative capability, dietary energy, multi-omics, lactating donkeys, milk components

## Abstract

This study was conducted to evaluate the effect of varying dietary energy levels on milk production, feed intake, nutrient digestion and metabolism, and antioxidation function of lactating donkeys, and integrating 16S rRNA gene sequencing, metabolomics, and proteomics to comprehensively reveal the underlying regulatory networks. A single-factor, completely randomized design was used in this study. Twenty-four Dezhou donkeys with similar milk yield (3.25 ± 0.46 kg/d), lactation days (29 ± 4.34 d), parities (4.17 ± 1.17), and body weight (256 ± 34 kg) were randomly divided into three dietary treatments (*n* = 8), and either a fed high-energy diet (DE = 13.1 MJ/kg, HED), medium-energy diet (DE = 12.4 MJ/kg, MED), and low-energy diet (DE = 11.7 MJ/kg, LED). The experiment period included 2 weeks for adaptation and 8 weeks for data and sample collection. Orthogonal polynomial contrasts were used to evaluate the linear and quadratic effects of increasing dietary energy. There were no significant interaction effects between dietary energy level and lactation week on any milk production and quality variables (*p* > 0.05). Increasing dietary energy level increased DMI, milk production, milk production efficiency, and milk components (linear and quadratic; *p* < 0.05). Increasing dietary energy improved the digestibility of DM and neutral detergent fiber (linear; *p* < 0.05), and crude protein digestibility, energy digestibility and metabolism, and nitrogen metabolism (quadratic; *p* < 0.05). However, it decreased BHBA and NEFA concentrations (linear; *p* < 0.05). Furthermore, increasing dietary energy first increased then decreased the activities of GSH-PX, SOD, and T-AOC (linear and quadratic; *p* < 0.05), while increasing the MDA content (linear; *p* < 0.05). Compared with HED and MED, LED increased the relative abundance of the *genera unclassified_f_Syntrophomonadaceae*, *Christensenellaceae_R-7_group* and *Treponema_2*. Compared with HED, MED increased the relative abundance of the *genera Ruminiclostridium_5*, *Ruminiclostridium_1*, *Family_XIII_UCG-001*, *unclassified_o__Clostridiales* and *norank_f__PL-11B10*. Thyroid hormone synthesis, tyrosine metabolism, and glutathione metabolism pathways are critical metabolic routes; these pathways can enhance energy metabolism and antioxidant function, thereby improving the milk production performance of lactating donkeys. In conclusion, the digestible energy of 12.40 MJ/kg was optimal for the milk performance of lactating donkeys, whereas excessively high dietary energy (13.1 MJ/kg) may reduce milk performance.

## 1. Introduction

Donkey milk is a functional dairy product with antioxidant properties, beneficial for the human oxidative defense system, and has the potential to serve as an adjuvant in conventional therapies for treating cardiovascular diseases, metabolic disorders, and intestinal health [1]. Additionally, studies have shown that donkey milk has lower allergenicity and is more suitable for infants and young children [2,3], making it an ideal substitute for human milk. However, the donkey industry is an emerging sector that has developed in recent years, with relatively underdeveloped husbandry and management techniques, resulting in even lower milk production and milk quality [4]. Consequently, enhancing donkey milk yield and improving its quality represent urgent challenges to be addressed in the development of the donkey industry.

Milk production could be affected by season, environment, parity and dietary nutrient level. Research in dairy cows had indicated that milk performance was effectively improved by providing optimal dietary energy and protein [5]. Low dietary energy levels resulted in the negative energy balance of dairy cows [6], especially in early lactation when the cows required more energy to meet the production demands. β-hydroxybutyric acid (BHBA) and non-esterified fatty acids (NEFAs) are key indicators of energy metabolism and lipid mobilization in animals. Increased concentrations of BHBA and NEFA, along with decreased concentrations of glucose and insulin in blood plasma, indicate impaired metabolic status in animals [7]. With increasing dietary energy levels, the glucose, insulin and insulin-like growth factor 1 concentrations in the serum of sheep were increased, while NEFA was decreased [8]. Zhou et al. (2015) [9] suggested that apparent digestibility, milk yield, milk protein content and yield, and lactose yield of dairy cows were increased with the increasing dietary energy. The higher dietary energy improved the nutrient digestion, total antioxidant capacity, and superoxide dismutase, while the medium dietary energy had lower maleic dialdehyde of beef cow [10]. Jabbar et al. (2013) [11] found that the milk yield of dairy cows was enhanced with moderate dietary energy levels, whereas excessively high dietary energy levels resulted in a reduction in milk production. It indicated that dietary energy levels may affect nutrient digestion, metabolism, and antioxidant status, thereby influencing milk yield and quality, but the mechanisms and influence of dietary energy levels on milk yield and antioxidative capabilities in lactating donkeys had not yet been systematically investigated. Therefore, the effect of dietary energy level on milk yield and quality warrants investigation in lactating donkeys.

Variations in the composition of animal body fluids (blood, milk, and urine) served as indicators of physiological and metabolic changes [12], and these changes were critically linked to the milk performance of dairy animals [13]. However, these were usually focused on dairy cows, and studies in donkeys remain scarce. At present, microbial populations, metabolomics and proteomics are widely used to explain the mechanism of nutrients regulating animal production. Therefore, this study hypothesizes that optimal dietary energy can increase milk performance, nutrient digestion and metabolism, and antioxidation function of lactating donkeys, by altering their microbial, metabolite and protein composition. This study aims to determine the effects of different dietary energy levels on milk yield and antioxidation function of lactating donkeys, elucidate the underlying molecular mechanisms and key regulatory pathways, and identify associated potential biomarkers. These findings are expected to provide a theoretical basis for establishing appropriate dietary energy levels and enhancing milk yield and quality in lactating donkeys via nutritional interventions.

## 2. Materials and Methods

### 2.1. Animals, Experimental Design, and Diets

All procedures were approved by the Inner Mongolia Agricultural University Animal Care and Use Committee (protocol no. 2019035). A single-factor, completely randomized design was used in this study. Twenty-four lactating Dezhou donkeys with foals and similar parity (4.17 ± 1.17), body weight (256 ± 34 kg), and DIM (29 ± 4.34 d) were randomly assigned to three group and fed a higher energy diet (DE = 13.10 MJ/kg, HED), a medium-energy diet (DE = 12.40 MJ/kg, MED), and a low-energy diet (DE = 11.70 MJ/kg, LED). The experiment included 2 weeks for adaptation and 8 weeks for data and sample collection. The donkeys were fed the same diet during the adaptation period and were individually housed in a stall with their foal and fed twice daily in equal meals at 700 h and 1400 h, respectively. Feed and water were provided ad libitum during the study. The ingredient compositions of the diets are presented in Table 1. The diets were formulated according to the nutrient requirements of horses [14] (NRC, 2007). The diet forage to concentrate ratio was 30:70 (DM basis). The diets were offered as a total mixed ration and were freshly prepared twice daily.

### 2.2. Sample Collection

Although diets were expected to be entirely consumed, the amounts offered and orts were weighed daily for each donkey. Feed component and TMR samples were collected weekly for DM and chemical composition analysis. Foals were separated from 700 h to 1000 h and 1400 h to 1700 h. Milking was conducted twice daily at 1000 h and 1700 h using an individual vacuum pumping machine (JuduH5402, Judu Technology, Xingtai, China), and milk yield (kg/d) was recorded at each milking. Milk samples from the morning and afternoon were combined in a 1:1 ratio and obtained over 3 consecutive days each week. At least 50 mL blended sample was used for analyzing the milk fat, protein, lactose, total solids (TS), solids not fat (SNF), and milk urea-N (MUN) using infrared spectroscopy (AOAC, 2000, pages 31–162; method 972.160) [15]. by Michigan DHIA (MilkoScan F100; Foss Electric, Hillerød, Denmark). For the final week, 5 mL milk samples were rapidly frozen in liquid nitrogen for metabolomic analysis, while the 15 mL milk samples were centrifuged at 4 °C and 1000× *g* for 10 min to remove fat, followed by further centrifugation at 4 °C and 10,000× *g* for 30 min to remove casein. The whey fraction was aliquoted into cryovials and rapidly frozen in liquid nitrogen for proteomic analysis.

During the final week of the experiment, the rectal feces and urine from all donkeys were collected continuously over 5 consecutive days to determine nutrient digestibility. On the final day of the experiment, rectal feces specimens were collected for microbiome analysis. In total, 20 mL of blood was collected from the jugular vein in the morning before feeding, then centrifuged (3000× *g* for 20 min) to obtain serum, and frozen at 20 °C for analyzing biochemical indicators, hormones, and antioxidation indicators. Another serum was rapidly frozen in liquid nitrogen for metabolomic analysis.

### 2.3. Chemical Analysis and Calculations

Samples of diets and feces were dried in a forced-air oven at 65 °C for 48 h, and then ground through a 1 mm screen. The diets and feces were analyzed for DM (method 967.03), CP (method 984.13), ether extract (EE, method 920.39), calcium (Ca), and phosphorus (P, method 935.13) according to AOAC (2000) [15]. Neutral detergent fiber (NDF) and acid detergent fiber (ADF) were determined according to the methods described by Van Soest et al. (1991) [16] using a Fiber Analyser (ANKOM Technology, Macedon, NY, USA) with an amylase, without sulfite, and were corrected for ash. Acid-insoluble ash (AIA) was used to determine the apparent digestibility (AD) of a certain nutrient according to the description of Van Keulen and Young (1977) [17].

AD (%) = 100 − [(A1 × B)/(A × B1)] × 100, where A is the content of a given nutrient in the diet (%), A1 is the content of the same nutrient in the feces (%), B is the content of AIA in the diet (%), and B1 is the content of AIA in the feces (%).

Milk yield (kg/d) was recorded at each milking, and daily milk yield was calculated by summing the two measurements [18]. The estimated milk yield (EMY, kg/day) was calculated from milk yield measurements. Energy-corrected milk (ECM), solids-corrected milk (SCM), milk production efficiency, and milk protein synthesis efficiency were calculated according to the EMY and milk composition content. EMY (kg/d) = milk yield (kg/d)/separated time (h) × 24 h [19]. ECM (kg/d) = 0.327 × milk (kg/d) + 12.95 × fat (kg/d) + 7.65 × protein [9]. SCM (kg/d) = milk kg [(12.24 × fat % × 0.01) + (7.10 × protein % × 0.01) + (6.35 × lactose % × 0.01) − 0.0345]. Milk production efficiency was calculated as EMY/DMI, SCM/DMI, and ECM/DMI. Milk protein synthesis efficiency = EMY (kg/d) × milk protein content (%)/DMI (kg/d). The total amount of urine was calculated according to the daily creatinine as 24.05 mg/kg of BW [20]. The energy of diets, feces, and urine was measured for analysis of energy metabolism by an oxygen bomb calorimeter (PPAR CO., Ltd., Moline, IL, USA). Gross energy intake (GEI), feces energy (FE), and urine energy (UE) were calculated according to the amount of DMI, feces, and urine. Digestible energy (DE, MJ/d) = GEI (MJ/d) − FE (MJ/d), and metabolizable energy (ME, MJ/d) = GEI (MJ/d) − FE (MJ/d) − UE (MJ/d). Protein biological value (BV) = (intake N (kg) − fecal N (kg) − urinary N (kg))/intake N(kg) − fecal N (kg) × 100%; nitrogen metabolism (%) = CP digestibility (%) × BV.

Serum biochemical indicators and hormones were determined as follows. Concentrations of total protein (TP), albumin (ALB), blood urea nitrogen (BUN), glucose (GLU), total cholesterol (TC), total bilirubin (TBIL), aspartate aminotransferase (AST), alanine aminotransferase (ALT), alkaline phosphatase (ALP), triglyceride (TG), creatinine (CREA), high-density lipoprotein (HDL), low-density lipoprotein (LDL), β-hydroxybutyrate (BHBA), non-esterified fatty acids (NEFAs), calcium (Ca), and phosphorus (P) were determined with commercial kits (Lepu diagnostics Co., Ltd., Beijing) using an automatic biochemistry analyzer (Hitachi 7020, Tokyo, Japan). The concentrations of adiponectin (ADPN, CK-E73323), prolactin (PRL, CK-E73212), hydrocortisone (HYD, CK-E73325), insulin (INS, CK-E73322), glucagon (GC, CK-E73324), and leptin (LEP, CK-E73366) were measured by enzyme-linked immunosorbent assay (ELISA) kits (Ruixin Biotechnology, Co., Ltd., Quanzhou, Fujian, China). The experiments were performed strictly according to the manufacturer’s instructions. Concentrations of urine creatinine (CREA) were analyzed by creatinine assay kit (C011-2-1, Nanjing Jiancheng Bioengineer Co., Ltd., Nanjing, Jiangsu, China). The serum glutathione peroxidase (GPx), thioredoxin reductase (TrxR), malondialdehyde (MDA), superoxide dismutase (SOD), and total antioxidant capacity (T-AOC) were measured using the standard commercial kits (Sinouk Institute of Biological Technology, Beijing, China).

### 2.4. Rectal Microbiome Analysis

Total DNA was extracted from rectal feces samples using an E.Z.N.A. soil DNA kit (Omega Bio-Tek, Norcross, GA, USA) according to the manufacturer’s protocol. A sample of 0.5 g of rectal feces was transferred to a 2 mL tube that contained 0.5 g of sterile 0.1 mm zirconia beads (BioSpec, Bartlesville, OK, USA) and 1 mL of SLX-Mlus Buffer. Samples were bead-beaten using a smashed grinder (TL-48R, Wan Bai Biotechnology Co., Ltd., Shanghai, China), operating at 45 Hz for 250 s, and a DNA sample was obtained following the manufacturer’s instructions. DNA purity and concentration were detected with NanoDrop2000 (Thermo Fisher, New York, NY, USA), and DNA integrity was assessed by 1% agarose gel electrophoresis. Specific primers (338F, 5′-ACTCCTACGGGAGGCAGCAG-3′; 806R, and 5′-GGACTACHVGGGTWTCTAAT-3′) were used to amplify the V3–V4 region of the 16S rRNA gene by an ABI GeneAmp^®^ 9700 PCR thermocycler (ABI, Foster, CA, USA). The PCR system (20 μL) contains 5× FastPfu Buffer (4 μL), 2.5 mmol/L dNTPs (2 μL), forward primer (0.8 μL, 5 μmol/L), reverse primer (0.8 μL, 5 μmol/L), FastPfu Polymerase (0.4 μL), BSA (0.2 μL), 10 ng template DNA, and ddH_2_O. The PCR amplification of 16S rRNA gene was performed as follows: initial denaturation at 95 °C for 3 min, followed by 27 cycles of denaturing at 95 °C for 30 s, annealing at 55 °C for 30 s and extension at 72 °C for 45 s, single extension at 72 °C for 10 min, and ended at 4 °C. PCR reactions were performed in triplicate. PCR products obtained were identified by electrophoresis on an2% agarose gel, purified by a DNA gel extraction kit (Axygen Biosciences, Union City, CA, USA), and quantified by a fluorometer (Quantus Fluorometer, Promega, Madison, WI, USA). The amplification products were sequenced on the Illumina MiSeq platform by Majorbio Bio-Pharm Technology Co., Ltd. (Shanghai, China).

Bioinformatic analysis of the gut microbiota was carried out using the Majorbio Cloud platform (https://cloud.majorbio.com). Operational taxonomic units (OTUs) were clustered using UPARSE (version 7.0.1090, https://drive5.com/uparse/ (accessed on 20 February 2026)) at a 97% sequence similarity threshold level. Taxonomy classification of OUT representative sequences was performed using the RDP Classifier (version 2.2). Based on the OTU information, Alpha-diversity was analyzed with Mothur (https://mothur.org/wiki/download_mothur/ (accessed on 20 February 2026)). Venn diagrams were generated in R (version 3.3.1). β-diversity analysis was conducted using QIIME (https://qiime.org/install/index.html (accessed on 20 February 2026)). Community composition analysis, test of significant difference between groups (phylum, family, and genus levels), linear discriminant analysis effect size (LEfSe), and correlation coefficients between environmental factors and remarkable genera (Spearman rank correlation coefficient) were calculated, and the resulting numerical matrix was presented through heatmaps. The raw sequencing data are available at the National Center for Biotechnology Information database (Bio Project ID: PRJNA1430129).

### 2.5. Untargeted Metabolomic Analysis

Metabolomic analysis of the plasma and milk samples was performed by liquid chromatography (LC) and mass spectrometry (MS) (Thermo Scientific, Waltham, MA, USA, Vanquish Horizon UHPLC System-Q Exactive HF-X) platform. The 100 μL samples were mixed with 400 μL of pre-cooled methanol: water (4:1, *v*/*v*) solution, and allowed to settle at −20 °C and treated by High throughput tissue crusher Wonbio-96c (Shanghai Wonbio Biotechnology Co., Ltd., Shanghai, China) at 50 Hz for 6 min, then followed by vortex for 30 s and ultrasound for 30 min (40 kHz, 5 °C). The samples were placed at −20 °C for 30 min to precipitate proteins. Samples were incubated at −20 °C for 30 min to precipitate proteins. Following centrifugation at 13,000× *g* and 4 °C for 15 min, the supernatant was carefully transferred to sample vials for LC-MS analysis.

The experimental procedures followed the standard protocol of Majorbio Bio-pharma Platform (Majorbio Bio-Pharma Technology Co., Ltd., Shanghai, China). The equal quantities of all sample metabolites were pooled to prepare quality control (QC) samples. During instrumental analysis, one QC sample was inserted into every three samples to assess the repeatability of the entire analytical process. Chromatographic separation was achieved using an ACQUITY UPLC HSS T3 column (100 mm × 2.1 mm in diameter, 1.8 μm; Waters, Milford, MA, USA). Two solutions were used as the mobile solvent: solvent A consists of 95% water and 5% acetonitrile (containing 0.1% formic acid), and solvent B consists of 47.5% acetonitrile, 47.5% isopropanol, and 5% water (containing 0.1% formic acid). The injection volume was 2 μL, and the column temperature was maintained at 40 °C. Mass spectrometry was carried out in positive- and negative-ion modes. Metabolite identification and screening were conducted by matching MS and MS/MS information with reliable biochemical databases, such as the Human Metabolome Database (HMDB) (http://www.hmdb.ca/) and Metlin database (https://metlin.scripps.edu/). Metabolites pathway analysis was performed on the Kyoto Encyclopedia of Genes and Genomes (KEGG) database (http://www.genome.jp/kegg/, (accessed on 20 February 2026)). Data analysis was performed using the Majorbio Cloud Platform (https://cloud.majorbio.com).

### 2.6. Whey Proteomic Analysis

Samples were lysed using an SDT buffer (4% SDS, 100 mM Tris-HCl, and 1 mM DTT, pH 7.6) for protein extraction. Protein concentration was determined with the Thermo Scientific Pierce BCA Assay Kit. Protein samples (100 μg) were diluted with triethylammonium bicarbonate (TEAB) buffer to a final concentration of 100 mmol/L. Tris(2-carboxyethyl) phosphine (TCEP) was added to a final concentration of 100 mmol/L, and the mixture was incubated at 37 °C for 60 min. Subsequently, iodoacetamide (IAM) was added to a final concentration of 40 mmol/L, followed by incubation at room temperature in the dark for 40 min. Pre-chilled acetone (acetone-to-sample volume ratio = 6:1) was added to each tube, and proteins were precipitated at −20 °C for 4 h. After centrifugation at 10,000× *g* for 20 min, the pellet was collected and fully dissolved in 100 μL of 100 mmol/L TEAB. The samples were then digested with trypsin at an enzyme-to-protein mass ratio of 1:50 at 37 °C overnight to generate peptides.

RPLC First-Dimension Separation: Peptides were reconstituted in UPLC loading buffer and subjected to high-pH liquid phase separation using a reversed-phase C18 column (ACQUITY UPLC BEH C18 Column, 1.7 μm, 2.1 mm × 150 mm, Waters, USA). Mobile phase A consisted of 2% acetonitrile (adjusted to pH 10 with ammonium hydroxide), and mobile phase B consisted of 80% acetonitrile (adjusted to pH 10 with ammonium hydroxide). The chromatography separation was performed on a Thermo Scientific Vanquish Flex binary UHPLC system with UV detection at 214 nm, a flow rate of 200 μL/min, and an elution time of 48 min. A total of 20 fractions were collected based on peak shape and elution time, which were subsequently pooled into 10 fractions and concentrated by vacuum centrifugation.

Liquid chromatography–tandem mass spectrometry (LC-MS/MS): The second-dimension analysis was performed using an upgraded liquid chromatography–tandem mass spectrometry system (Easy-nLC 1200 coupled with an Orbitrap Exploris 480 mass spectrometer, Thermo Fisher Scientific, New York, NY, USA). Peptides from the first-dimension RPLC separation were dissolved in MS loading buffer and loaded onto a C18 analytical column (75 μm × 25 cm, Thermo, USA) for separation over 120 min at a flow rate of 300 nL/min. Gradient elution was performed using the EASY-nLC system (Thermo Fisher Scientific, New York, NY, USA), with mobile phase A (2% acetonitrile with 0.1% formic acid) and mobile phase B (80% acetonitrile with 0.1% formic acid). MS scanning parameters were as follows: scan range (*m*/*z*): 350–1500; acquisition mode: data-dependent acquisition (DDA); cycle time: 2 s; full MS resolution: 120,000; maximum injection time: 20 ms; fragmentation mode: higher-energy collisional dissociation (HCD); MS/MS resolution: 45,000; maximum injection time: 86 ms; fixed first mass: 110 *m*/*z*; minimum automatic gain control (AGC) target: 8 × 10^3^; intensity threshold: 8.3 × 10^4^; and dynamic exclusion time: 40 s.

The raw mass spectrometry data were processed and analyzed using Proteome Discoverer™ Software 2.4. Database searching was performed against the UniProtKB Equus database (https://www.uniprot.org/taxonomy/9789 (accessed on 20 February 2026)). During the search, the peptide-spectrum match false discovery rate (FDR) was set at ≤0.01, and a minimum of one unique peptide was required for protein identification.

Bioinformatic analysis of proteomic data was performed with the Majorbio Cloud platform (https://cloud.majorbio.com). Hierarchical clustering of both samples and protein expression levels was performed using the Complex heatmap package (R Version 3.4) to generate a clustered heatmap. Functional enrichment analysis was conducted using Fisher’s exact test to compare the distribution of Gene Ontology (GO) terms (http://geneontology.org/) or KEGG pathways (http://www.genome.jp/kegg/ (accessed on 20 February 2026)) between the target protein set and the entire identified proteome. GO annotation and KEGG pathway enrichment analyses were performed accordingly. Protein–protein interaction (PPI) networks were constructed based on information retrieved from the STRING database (http://string-db.org/). The interaction network was generated and subsequently analyzed to explore functional associations among the target proteins.

Based on the KEGG pathway analysis results of differential metabolites and proteins, those co-participating in the same pathway were screened. The identified differential metabolites and proteins within each pathway were then mapped to the KEGG database to generate integrated pathway diagrams, illustrating the combined roles of proteins and metabolites.

### 2.7. Statistical Analysis

Experimental data were collated and processed using Microsoft Excel 97–2003. All statistical analyses were performed using SAS software (version 9.4, SAS Institute Inc., Cary, NC, USA). Treatment effects of DMI, milk yield, milk component yield, milk composition, and feed efficiency were analyzed using the PROC MIXED procedure, with lactation week treated as a repeated measure using an autoregressive covariance structure:

Yijk = μ + Ci + Wj + CWij + bXjk + εijk,

where Yijk = dependent variable,μ = overall mean, Ci = fixed effect of dietary energy level; Wj = fixed effect of lactation week (weeks 1, 2, 3, 4, 5, 6, 7, and 8), CWij = effect of the interaction between dietary energy and lactation week, bXjk = effect of covariate (week 0, the observations during the 2 weeks of pretrial period served as covariates for the corresponding experimental period), and εijk = residual error. The interactions of Ci × Wj were initially included in the model and removed when not significant.

Orthogonal polynomial contrasts were used to detect linear and quadratic responses to dietary energy for all response variables. Statistically significant differences were declared at *p* < 0.05, with trends noted at 0.05 < *p* ≤ 0.10.

Kruskal–Wallis H test sums were utilized to analyze differences among phyla, families, and genera of the rectal bacteria. Additionally, the rectal microbial abundance of Jennies at the genus level was assessed using Linear Discriminant Analysis Effect Size (LEfSe), with a threshold LDA score exceeding 3 for significance. Differentially expressed metabolites were screened based on a fold change (FC) > 1 and *p* < 0.05. For whey proteomics, Student’s *t*-test was applied to calculate the significance (*p*-value) of differential expression between sample groups, along with the fold change (FC). Differentially expressed proteins were identified using the following criteria: proteins with *p* < 0.05 and FC > 1.2 were classified as upregulated, while those with *p* < 0.05 and FC < 0.83 were classified as downregulated.

## 3. Results

### 3.1. Dry Matter Intake and Milk Performance

As shown in Table 2, there were no significant interaction effects between dietary energy level and lactation week on any milk production or quality variables (*p* > 0.05). Therefore, the main effects of dietary energy levels are presented based on orthogonal polynomial contrasts. Increasing dietary energy level increased and then decreased DMI milk yield, EMY, ECM, SCM, milk production efficiency (EMY/DMI, ECM/DMI, SCM/DMI, and milk protein synthesis efficiency), milk components (fat, protein, lactose, and TS), and milk component yields (fat, protein, lactose, SNF, and TS) (*p* < 0.05), with the highest values observed in the MED group. Increasing dietary energy decreased and then increased MUN component (quadratic; *p* = 0.002), with the lowest value observed in the MED group.

### 3.2. Nutrient Digestibility, Energy Metabolism, and Nitrogen Metabolism

As shown in Table 3, increasing dietary energy increased the digestibility of DM, EE and NDF (linear; *p* < 0.05), with the highest values observed in the HED group. Increasing dietary energy decreased the digestibility of Ca and P (linear; *p* < 0.05). The digestibility of CP, ADF, and energy, as well as energy metabolism and BV, were linearly and quadratically increased with increasing dietary energy (*p* < 0.05). Increasing dietary energy increased then decreased nitrogen metabolism (quadratic; *p* < 0.05), with the highest values being observed in the MED group.

### 3.3. Serum Biochemical Parameters, Hormones, and Antioxidative Index

As shown in Table 4, the serum BUN concentration increased both linearly and quadratically with increasing dietary energy (*p* < 0.05), with the lowest value observed in the MED group. Increasing dietary energy also increased AST, ALP, TG, CHO, LDL-C, and Glu (linear; *p* < 0.05), with the highest value observed in the HED group. Increasing dietary energy increased ALT and HDL-C (quadratic; *p* < 0.05), but decreased BHBA and NEFA concentrations (linear; *p* < 0.05), with the lowest value observed in the HED group. The serum concentrations of TP, ALB, and TBIL were not affected by dietary energy level (*p* > 0.05).

Data on serum hormones and antioxidant indexes are shown in Table 5. Increasing dietary energy increased and then decreased serum concentrations of PRL, INS, and LEP (quadratic; *p* < 0.05), and increased HYD (quadratic; *p* = 0.012), but decreased and then increased ADPN concentration (linear and quadratic; *p* < 0.05) and tended to decrease GC (linear; *p* = 0.067). Increasing dietary energy increased and then decreased the activities of GSH-PX (quadratic; *p* = 0.001), SOD (quadratic; *p* = 0.004), and T-AOC (linear and quadratic; *p* < 0.05) with the highest value being observed in the MED group, and increased MDA (linear; *p* = 0.011) with the highest value observed in the HED group.

### 3.4. The Diversity of Rectal Microbiota in Lactating Donkeys

As shown in Table 6, according to the α-diversity analysis, community coverage reached 98% for all groups, indicating that rectal microorganism species and structural diversity could be accurately assessed. Compared with the HED group, the Sobs, ACE, Chao, and Shannon indices were higher, while Simpson was lower in the MED and LED groups (*p* < 0.05).

**Figure 1:**
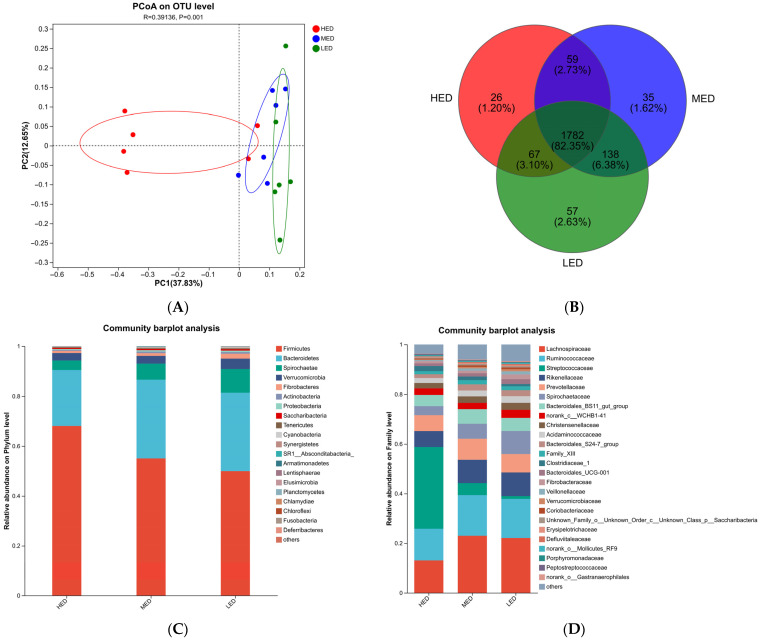
Differential bacterial compositional functions among HED, MED, and MED. The principal coordinate analysis (PCoA) (**A**) and VENN diagram of rectal microorganisms in three groups (OUT level) (**B**). The relative abundances of rectal bacteria are at the phylum (**C**) and family levels (**D**).

Principal coordinate analysis (PCoA) based on the Bray-Curtis distance matrix revealed that the HED rectal microbial samples had more variation than in the MED and LED groups, with PC1 and PC2 accounting for 37.83% and 12.65% of the total variation, respectively (Figure 1A), indicating that there were differences in rectal microbial species. The number of shared and unique species across several groups or samples can be counted using Venn diagrams. In this experiment, non-repeated sequences were clustered using operational taxonomic units (OTU) based on 82.35% similarity, which produced 2166 OTU. Of these, 26,35 and 57 unique OTU were found in the HED, MED, and LED groups, respectively, making a total of 1782 OTU among the three groups (Figure 1B).

### 3.5. Different Rectal Bacteria Among HDE, MED and LED Groups

Rectal microorganisms were analyzed at the phylum and family levels. At the phylum level, *Firmicutes* and *Bacteroidetes* were the dominant species in the rectal flora, making up more than 98% of all species (Figure 1C). The relative abundance was 68.02% and 22.47% in HED, 54.92% and 31.72% in MED, 49.93% and 31.48% in LED, respectively. At the family level, the dominant families were *Streptococcaceae*, *Lachnospiraceae*, *Ruminococcaceae*, *Prevotellaceae*, and *Rikenellaceae*. Within the HED group, the relative abundances were 32.86%, 13.08%, 12.74%, 6.38%, and 6.37%, respectively. The MED group were 4.80%, 22.96%, 16.32%, 8.50%, and 9.48%, respectively. The LED group had relative abundances of 1.13%, 22.13%, 15.75%, 7.42%, and 9.58%, respectively (Figure 1D).

**Figure 2:**
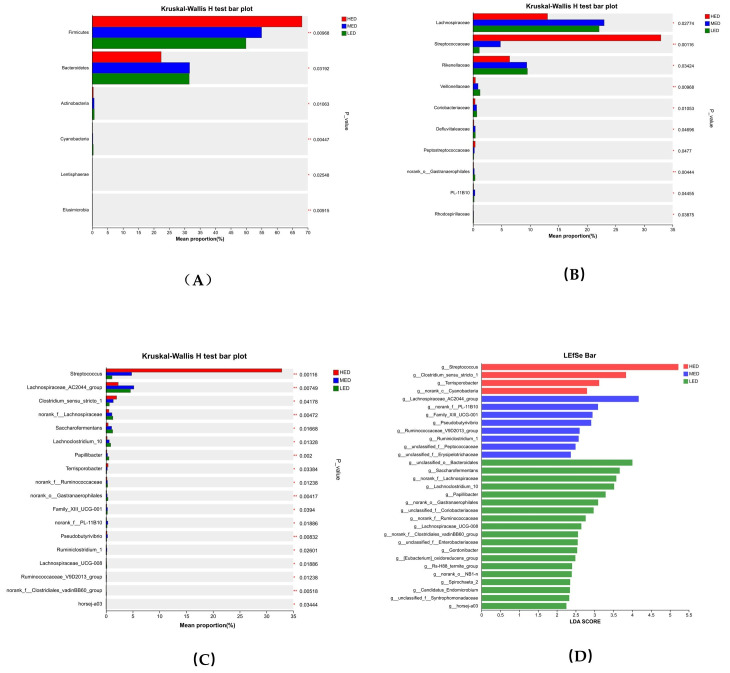
Differential bacterial compositions at the (**A**) phylum, (**B**) family, and (**C**) genus levels using 16S rRNA sequence data based on the Kruskal–Wallis H test. LEfSe analysis of rectal microbiota among 3 treatments (**D**). Linear discriminant analysis (LDA) value distributed histogram, and the score ≥ 3 means significant. HED =high-energy diet, MED = medium-energy diet, LED = low-energy diet. * *p* < 0.05 and ** *p* < 0.01.

Changes in microbial communities at the phylum, family, and genus levels in the three groups are shown in Figure 2. At the phylum level (Figure 2A), the relative abundance of *Firmicutes* was higher in the HED group than that in the MED and LED groups, and *Bacteroidetes* was lower (*p* < 0.05). The relative abundance of *Spirochaetae* was lower in the HED and MED groups than in the LED group (*p* < 0.05). The abundance of *Verrucomicrobia* and *Fibrobacteres* was not different among the three groups (*p* > 0.05). At the family level (Figure 2B), the relative abundance of *Streptococcaceae* was lower in MED and LED groups. In contrast, the abundance of *Lachnospiraceae*, *Rikenellaceae* and *Coriobacteriaceae* was higher in the MED and LED groups (*p* < 0.05). The abundance of *unclassified_p__Bacteroidetes* was greater in MED group than that in HED and LED groups (*p* < 0.05). There was no difference in *Ruminococcaceae* and *Prevotellaceae* abundance among the three groups (*p* > 0.05). At the genus level (Figure 2C), compared with MED and LED groups, *Streptococcus* abundance was greater in the HED group (*p* < 0.05), but the abundance of *nclassified_f__Lachnospiraceae*, *Lachnospiraceae_AC2044_group*, *Lachnospiraceae_XPB1014_group*, *Ruminococcaceae_UCG-010*, *Prevotella_1* and *Lachnospiraceae_UCG-009* were lower (*p* < 0.05). Compared with LED group, the abundance of *norank_f__Bacteroidales_S24-7_group* abundance was higher in the HED and MED groups, and the abundance of *unclassified_o__Bacteroidales*, *Rikenellaceae_RC9_gut_group*, *norank_f__Bacteroidales_UCG-001* were lower in the HED group (*p* < 0.05).

A LEfSe analysis was performed to identify different bacterial compositional differences among the 3 groups of rectal in lactating donkeys (Figure 2D). Figure 2,Compared with MED and LED groups, the HED increased the abundance of *Streptococcus*, *Clostridium_sensu_stricto_1* and *Terrisporobacter* (*p* < 0.05). Compared with HED and LED groups, the MED group increased the abundance of *Pseudobutyrivibrio*, *Lachnospiraceae_AC2044_group*, *Ruminiclostridium_5*, *Ruminiclostridium_1*, *Candidatus_Endomicrobium*, *Family_XIII_UCG-001*, *norank_f__Rhodospirillaceae*, *norank_f__PL-11B10*, *unclassified_f__Erysipelotrichaceae*, *Ruminococcaceae_V9D2013_group*, *and unclassified_o__Clostridiales* (*p* < 0.05). Compared with HED and MED groups, the LED increased the abundance of *horsej-a03*, *unclassified_o__Bacteroidales*, *norank_f__Ruminococcaceae*, *Quinella*, *Papillibacter*, *[Eubacterium]_oxidoreducens_group*, *Spirochaeta_2*, *Lachnoclostridium_10*, *unclassified_c__Alphaproteobacteria*, *Lachnospiraceae_UCG-008*, *unclassified_f__Syntrophomonadaceae*, *unclassified_f__Coriobacteriaceae*, *norank_o__Gastranaerophilales*, *norank_f__Clostridiales_vadinBB60_group*, *Defluviitaleaceae_UCG-011*, *norank_f__Lachnospiraceae*, and *Saccharofermentans* (*p* < 0.05).

### 3.6. Correlation Analysis of Different Rectal Bacterial Genera with Milk Performance and Nutrient Digestibility

**Figure 3:**
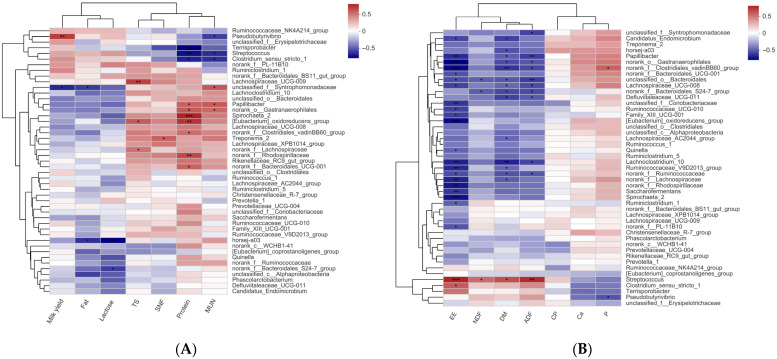
The Spearman correlation analysis between milk performance and rectal bacteria (**A**). The Spearman correlation analysis between nutrient digestibility and rectal bacteria (**B**). * *p* < 0.05, ** *p* < 0.01 and *** *p* < 0.001.

A correlation heatmap was constructed using Spearman’s correlation coefficient to examine the relationship between milk performance and the digestibility of nutrients (Figure 3A). According to the results, milk yield was positively correlated with *Pseudobutyrivibrio*, and was negatively correlated with *unclassified_f__Syntrophomonadaceae*. Milk fat content was negatively correlated with *horsej-a03* and *unclassified_f__Syntrophomonadaceae*. Lactose content was negatively correlated with *horsej-a03* and *norank_f__Bacteroidales_S24-7_group*. TS content was positively correlated with *Lachnospiraceae_UCG-009*, *[Eubacterium]_oxidoreducens_group*, and *norank_f__Lachnospiraceae*. SNF content was positively correlated with *Treponema_2*. Milk protein content was positively correlated with *Spirochaeta_2*, *[Eubacterium]_oxidoreducens_group*, *norank_f__Rhodospirillaceae*, *norank_o__Gastranaerophilales*, *Papillibacter*, *norank_f__Bacteroidales_UCG-001*, *and norank_f__Clostridiales_vadinBB60_group*. The MUN was positively correlated with *unclassified_f__Syntrophomonadaceae*, *Papillibacter*, and *norank_o__Gastranaerophilales*, and negatively correlated with *Pseudobutyrivibrio*, *Streptococcus*, and *Clostridiales sensu stricto_1*.

The digestibility of DM, EE, NDF, and ADF of lactating donkeys was positively correlated with *Streptococcus* (Figure 3B). But the digestibility of DM and ADF was negatively correlated with *Lachnospiraceae_UCG-008*, *Papillibacter*, *unclassified_o__Bacteroidales*, *norank_f__Ruminococcaceae*, *Lachnoclostridium_10*, *norank_o__Gastranaerophilales*, *norank_f__Bacteroidales_S24-7_group*, *Defluviitaleaceae_UCG-011*, and *norank_f__Clostridiales_vadinBB60_group*. While digestibility of DM was negatively correlated with *Ruminococcaceae_V9D2013_group*, *Candidatus_Endomicrobium*, *horsej-a03*, *Lachnospiraceae_AC2044_group*, and *norank_f__Lachnospiraceae*. NDF digestibility was negatively correlated with *unclassified_o__Bacteroidales*, and *norank_f__Bacteroidales_S24-7_group*. EE digestibility was negatively correlated with *Ruminiclostridium_1*, *Quinella*, *norank_f__PL-11B10*, *Lachnoclostridium_10*, *Family_XIII_UCG-001*, *norank_f__Bacteroidales_UCG-001*, *Papillibacter*, *norank_f__Ruminococcaceae*, *Candidatus_Endomicrobium*, *Lachnospiraceae_UCG-008*, *Ruminococcaceae_UCG-010*, *Saccharofermentans*, *unclassified_f__Coriobacteriaceae*, *Spirochaeta_2*, *Ruminococcaceae_V9D2013_group*, *norank_f__Clostridiales_vadinBB60_group*, *norank_f_Lachnospiraceae*, *norank_f_Rhodospirillaceae*, *norank_o-Gastranaerophilales*, and *[Eubacterium]_oxidoreducens_group*.

### 3.7. Metabolomic Profiles in Serum and Identification of Metabolites

**Figure 4:**
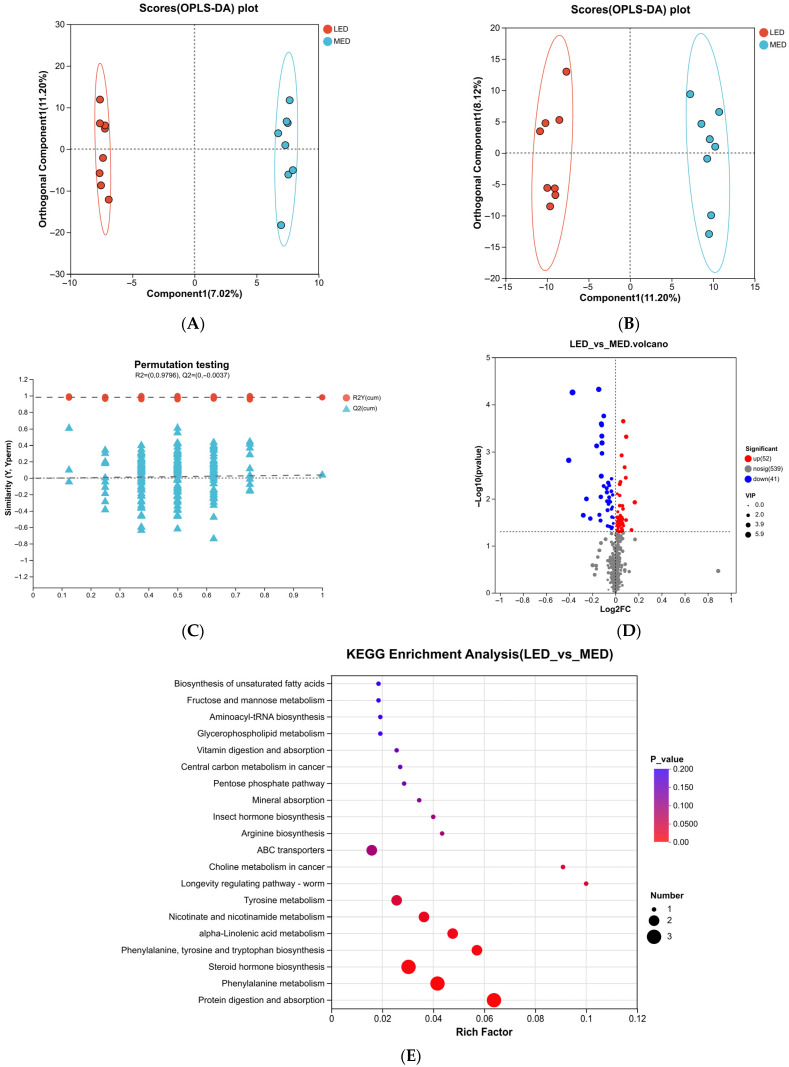
Serum metabolome profiles of LED vs. MED. (**A**,**B**) Orthogonal partial least squares discrimination analysis (OPLS-DA). (**C**) OPLS-DA substitution test. (**D**) The volcano plots of serum differential metabolites. (**E**) Metabolic pathway enrichment analysis of the differentially presented compounds between MED and LED groups. MED = medium-energy diet, LED = low-energy diet.

The OPLS-DA analysis results are presented in Figure 4A,B, with the corresponding permutation test results shown in Figure 4C. Figure 4A,B displays the OPLS-DA score plots comparing the LED and MED groups in positive and negative ionization modes, respectively. As shown, the serum metabolite profiles of lactating donkeys exhibited clear separation between the LED vs. MED, with no overlap observed in either ionization mode. The validity of the OPLS-DA model was primarily indicated by the parameters R2Y and Q2, while the permutation test was used to evaluate the model’s rationality and reliability. The R2Y values for the OPLS-DA model were 0.980, indicating a strong fit of the model to the observed data. As shown in Figure 4D, a total of 93 differential metabolites (DMs) were identified in the LED vs. MED. Among these DMs, 52 were significantly upregulated, and 41 were significantly downregulated.

For the LED vs. MED comparison, the 93 differentially abundant metabolites identified in serum were subjected to metabolic pathway enrichment analysis using MetPA. The results indicated significant enrichment in the phenylalanine metabolism pathway, phenylalanine, tyrosine, and tryptophan biosynthesis pathway, tyrosine metabolism, and the nicotinate and nicotinamide metabolism pathway were enriched (Figure 4E, Table 7).

As shown in Table 8, it was shown that the hydrocinnamic acid was significantly negatively correlated with both NEFA and BHBA, while L-Phenylalanine showed significant positive correlations with both NEFA and BHBA. Dihydro-3-coumaric acid showed a significant negative correlation with BHBA.

### 3.8. Metabolomic Profiles in the Milk and Identification of Metabolites

**Figure 5:**
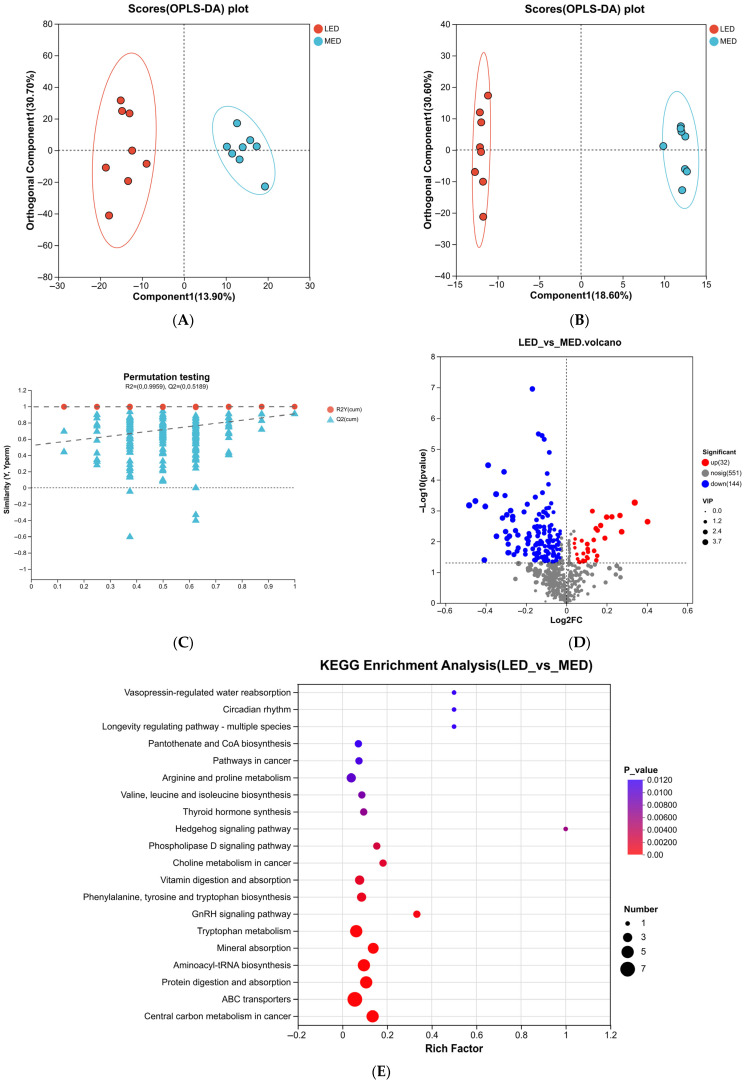
Milk metabolome profiles of LED vs. MED. (**A**,**B**) The orthogonal partial least squares discrimination analysis (OPLS-DA) following positive ion electrospray ionization (ESI+) and negative ion electrospray ionization (ESI−) modes, respectively. (**C**) The OPLS-DA substitution test. (**D**) The volcano plots of milk differential metabolites. (**E**) The metabolic pathway enrichment analysis of the differentially presented compounds between MED and LED groups. MED = medium-energy diet, LED = low-energy diet.

The results of OPLS-DA analysis for donkey milk are presented in Figure 5A,B, with the corresponding permutation test results shown in Figure 5C. Figure 5A,B displays the OPLS-DA score plots comparing the LED and MED groups in positive and negative ionization modes, respectively. Clear separation was observed between the groups in both modes, with no sample overlap. The validity of the OPLS-DA model was reflected by the parameters R2Y and Q2, whereas the permutation test was used to evaluate its reliability and predictive ability. The R2Y values for the models were 0.995, indicating a strong goodness of fit. The corresponding Q2 values were 0.519, exceeding 0.5, which suggested that the model was stable, reliable, and possesses a good predictive power. As illustrated in Figure 5D, a total of 176 differential metabolites (DMs) were identified in donkey milk in the LED vs. MED comparison. Overall, 32 metabolites were significantly upregulated, while 144 were significantly downregulated.

For the LED vs. MED comparison, the 176 differentially abundant metabolites identified in donkey milk were subjected to metabolic pathway enrichment analysis (MetPA). The analysis revealed significant enrichment in several metabolic pathways, including tryptophan metabolism, phenylalanine, tyrosine and tryptophan biosynthesis, pantothenate and CoA biosynthesis, arginine and proline metabolism, histidine metabolism, glycine, serine and threonine metabolism, glutathione metabolism, biotin metabolism, ascorbate, and aldarate metabolism (Figure 5E, Table 9).

As shown in Table 10, Niacinamide was significantly negatively correlated with serum BHBA levels (*p* = 0.044), and L-Tryptophan, D-Glucarate, and Biotin showed trends toward negative correlations with serum BHBA (*p* < 0.05). L-Serine and Tyramine were significantly positively correlated with serum NEFA, and L-Tyrosine showed a trend toward a significant positive correlation with serum NEFA. Glutathione and 3-Methyl-L-histidine were significantly negatively correlated with both serum NEFA and BHBA levels, and GDP-L-fucose was significantly negatively correlated with serum NEFA levels (*p* < 0.05).

### 3.9. Comprehensive Characterization of the Whey Proteome

**Figure 6:**
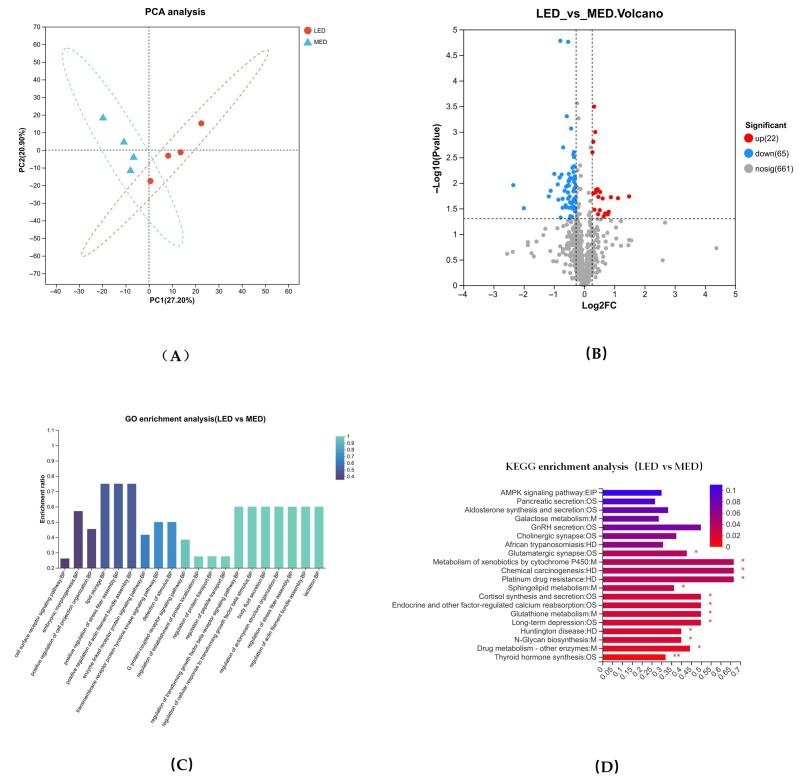
Milk protein profiles of LED vs. MED. (**A**) PCA in LED_vs_MED. (**B**) The volcano plots of milk differential whey proteins. (**C**) GO function enrichment analysis of the LED vs. MED group (top-20 category). The top 20 Gene Ontology (GO) terms of differentially synthesized proteins in the whey biopsies of lactating donkeys during (*n* = 4). Red bars represent biological process terms, blue bars represent cellular component terms, and green bars represent molecular function terms. (**D**) KEGG enrichment analysis (LED_vs_MED). MED = medium-energy diet and LED = low-energy diet. * *p* < 0.05 and ** *p* < 0.01.

Figure 6A presents the PCA analysis results of donkey milk proteins from the LED vs. MED comparison, showing that samples within each group are relatively concentrated and well-separated between groups. As shown in Figure 6B, a total of 87 differentially expressed proteins were detected between the LED and MED groups. Compared with the MED group, the LED group exhibited 22 upregulated and 65 downregulated proteins.

By enrichment of 87 differentially expressed proteins were functionally classified into 100 categories, with the top 20 categories all being biological processes (Figure 6C). KEGG pathway enrichment analysis was performed on a subset of the differentially expressed proteins from donkey milk in the LED vs. MED, revealing enrichment in 197 pathways. The top 20 enriched pathways were shown in Figure 6D, among which 13 were significant. These included glutamatergic synapse, sphingolipid metabolism, platinum drug resistance, chemical carcinogenesis, metabolism of xenobiotics by cytochrome P450, glutathione metabolism, endocrine and other factor-regulated calcium reabsorption, cortisol synthesis and secretion, long-term depression, thyroid hormone synthesis, drug metabolism—other enzymes, Huntington’s disease, and N-Glycan biosynthesis. Additionally, six pathways showed a trend toward significance. These include African trypanosomiasis, cholinergic synapse, GnRH secretion, galactose metabolism, aldosterone synthesis and secretion, and pancreatic secretion. In Table 11, it is shown that enrichment partial KEGG pathways of LED vs. MED. The thyroid hormone synthesis, glutathione metabolism, galactose metabolism, and PI3K-Akt signaling pathway were significantly enriched.

**Figure 7:**
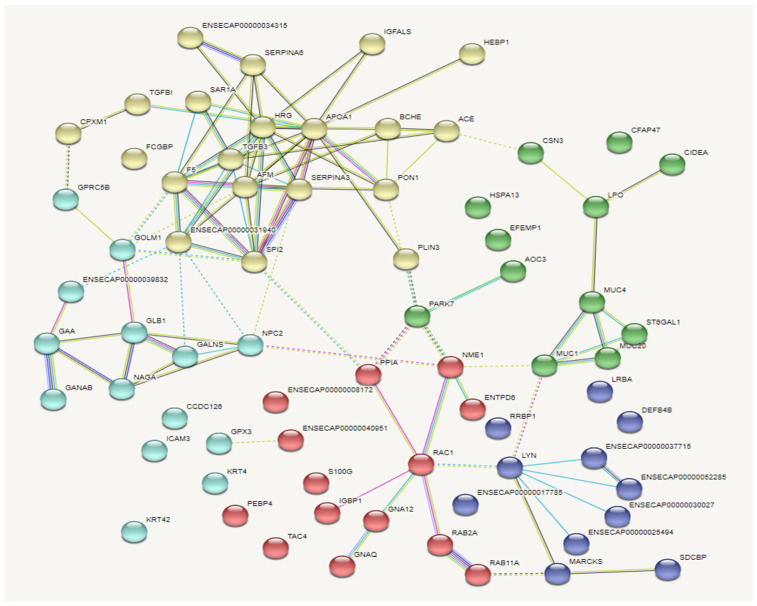
Protein–protein interaction (PPI) network analysis. TGFB3 = transforming growth factor beta-3 proprotein, AFM = afamin, HRG = histidine rich glycoprotein, APOA1 = apolipoprotein A1, SERPIN3 = serpin family A member, SERPIN6 = serpin family A member 6, TGFBI = transforming growth factor-beta-induced protein ig-h3, PON1 = paraoxonase 1, MUC1 = Mucin 1, MUC20 = Mucin 20, MUC4 = Mucin 4,ST6GAL1 = ST6 beta-galactoside alpha-2,6-sialyltransferase 1, LPO = Lactoperoxidase, GLB1 = beta-galactosidase, GAA = glucosidase alpha acid, NAGA = alpha-galactosidase, GALNS = N-acetylgalactosamine-6-sulfatase, GOLM1 = golgi membrane protein 1, LYN = tyrosine-protein kinase, RAC1 = rac family small GTPase 1, NME = nucleoside diphosphate kinase, and PPIA = peptidyl-prolyl cis-trans isomerase.

Figure 7 displayed the protein–protein interaction network among the differentially expressed proteins in the LED vs. MED, with the proteins categorized into five groups. In the LED vs. MED, the key interacting proteins in each cluster were as follows: in the green cluster, strongly associated proteins included Mucin 1 (MUC1), Mucin 20 (MUC20), Mucin 4 (MUC4), ST6 Beta-galactoside alpha-2,6-sialyltransferase 1 (ST6GAL1), Lactoperoxidase (LPO), and cell death-inducing DFFA-like effector a (CIDEA). In the blue cluster, the strongly associated protein was Tyrosine-protein kinase (LYN). In the red cluster, strongly associated proteins included Rac family small GTPase 1 (RAC1), Nucleoside diphosphate kinase (NME), Peptidylprolyl isomerase A (PPIA), and RAB2A, a member of the RAS oncogene family (RAB2A). In the cyan cluster, strongly associated proteins included Beta-galactosidase (GLB1), Glucosidase alpha acid (GAA), Alpha-N-acetylgalactosaminidase (NAGA), N-acetylgalactosamine-6-sulfatase (GALNS), and Golgi membrane protein 1 (GOLM1). In the yellow cluster, strongly associated proteins included Transforming growth factor beta-3 (TGFB3), Afamin (AFM), Histidine-rich glycoprotein (HRG), Apolipoprotein A1 (APOA1), Serpin family A member 3 (SERPIN3), Serpin family A member 6 (SERPIN6), Transforming growth factor beta-induced protein ig-h3 (TGFBI), and Paraoxonase 1 (PON1).

### 3.10. Integrated KEGG Pathway Analysis of Differentially Expressed Proteins and Differential Metabolites

**Figure 8:**
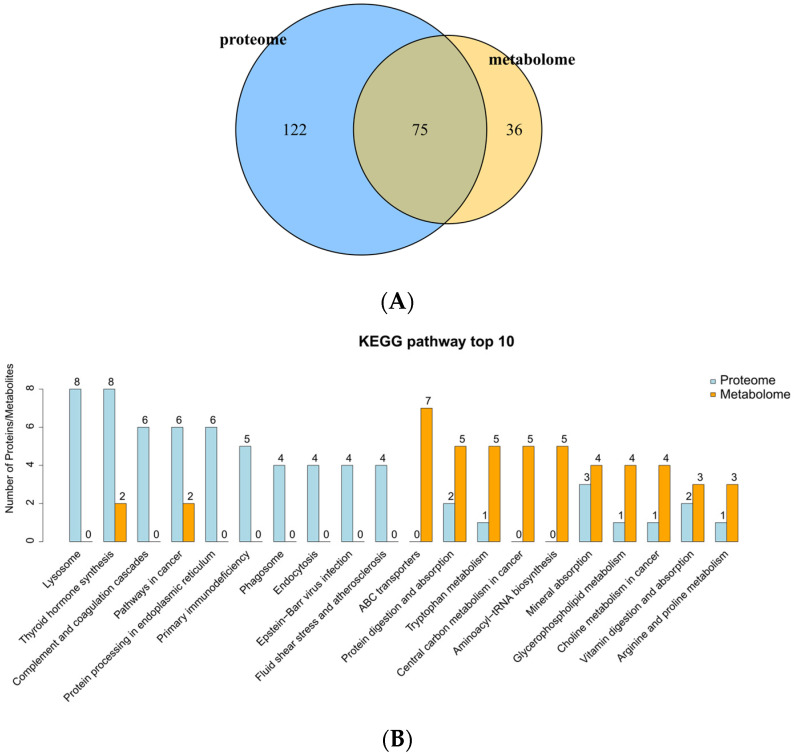
The number of pathways involved by differential proteins and differential metabolites in LED vs. MED (**A**). The top 10 KEGG pathways involved by differential proteins and metabolites in LED vs. MED (**B**). MED = medium-energy diet, LED = low-energy diet.

In the LED vs. MED, the pathways involved differentially expressed proteins from the proteome and differential metabolites from the metabolome were integrated to identify metabolic pathways and their counts co-enriched in both omics datasets. As shown in Figure 8A, the differentially expressed proteins were associated with 197 pathways, the differential metabolites with 111 pathways, and the pathways co-enriched in both omics were 75 pathways. As shown in Figure 8B, the top 10 KEGG pathways included both differentially expressed proteins and differential metabolites in the LED vs. MED. The co-enriched pathways included thyroid hormone synthesis, cancer pathways, protein degradation and absorption, tryptophan metabolism, mineral absorption, glycerophospholipid metabolism, choline metabolism in cancer, vitamin degradation and absorption, and arginine and proline metabolism.

In the LED vs. MED, 73 pathways were co-enriched with both differentially expressed proteins and differential metabolites. Some of the co-enriched pathways are presented in Table 12. Among them, the thyroid hormone synthesis pathway reached significant levels in both the proteomic and metabolomic analyses. Several pathways were specifically significant in the metabolomic analysis, including vitamin degradation and absorption, tryptophan metabolism, protein degradation and absorption, phospholipase D signaling pathway, and mineral absorption. The glutathione metabolism and glutamatergic synapse pathways were in the proteomic.

In the thyroid hormone synthesis, the differentially expressed protein serpin domain-containing protein (A0A3Q2HM80) was higher in LED than in the MED group, while lactoperoxidase (LPO and F7C3Z3), serpin family A member 6 (SERPIN6 and F7DRS2), vitamin D-binding protein (AFM and A0A3Q2ID60), guanine nucleotide-binding protein subunit alpha-12 (GAN12 and A0A3Q2H8B8), glutathione peroxidase (GSH-Px and A0A5F5PST7), guanine nucleotide-binding protein subunit alpha-q (GANQ and A0A3Q2IAY3), serpin family E member 3 (SERPIN3 and F6ZLR1), and the differential metabolites glutathione (GSH) and cyclic adenosine monophosphate (cAMP) were lower in LED than those in MED group.

In tyrosine metabolism, the differentially expressed proteins lactoperoxidase (LPO, and F7C3Z3) and amine oxidase 3 (AOC3 and F7DLT3), and the differential metabolite 4-hydroxyphenylpyruvate were lower in the LED group than those in the MED group. In the glutathione metabolism pathway, glutathione S-transferase (GST and A0A5F5PSQ4), glutathione peroxidase (GSH-Px and A0A5F5PST7), glutathione S-transferase alpha 1 (GSTA1 and M9ZUR8), and the differential metabolite GSH were lower in the LED group compared with the MED group.

In glycine–serine–threonine metabolism, the level of betaine in the LED group was higher, while the differential protein amine oxidase 3 (AOC3 and F7DLT3) and the differential metabolite L-tryptophan were lower than those of the MED group. In vitamin degradation and absorption metabolism, scavenger receptor class B type 1 (SR-B1 and A0A3Q2I5H0), apolipoprotein A1 (APOA1 and F6Z2L5), pantothenic acid, biotin, and nicotinamide were lower in the LED group than in the MED group. In protein degradation and absorption, the differential protein proton-coupled amino acid transporter (LOC111767478 and A0A5F5PQX9), angiotensin-converting enzyme (ACE and F6ZSR4), and the differential metabolites L-valine, L-histidine, L-tryptophan, L-proline, and L-isoleucine were lower in the LED group compared with the MED group. In histidine metabolism, the differential protein amine oxidase 3 (AOC3 and F7DLT3) and the differential metabolites L-histidine (L-His) and 3-methyl-L-histidine were lower in the LED group than in the MED group. In tryptophan metabolism, the level of indoleacetaldehyde in the LED group was higher than in the MED group, while amine oxidase 3 (AOC3 and F7DLT3), tryptophol, N-acetylserotonin, 2-aminobenzoic acid, and L-tryptophan (L-Trp) were lower compared to the MED group.

## 4. Discussion

### 4.1. The Effect of Dietary Energy on Milk Performance of Lactating Donkeys

Energy is a critical nutrient that affects animal production performance. Adequate dietary energy helps lactating animals prevent negative energy balance and other metabolic disorders [21]. Mustafa et al. (2017) [22] found that when buffaloes were fed diets with metabolizable energy levels of 10.93 MJ/kg, 12.17 MJ/kg, and 13.03 MJ/kg, the diets containing 12.17 MJ/kg and 13.03 MJ/kg significantly increased fat-corrected milk yield and feed conversion efficiency, with the highest values observed in the 12.17 MJ/kg group. Similarly, Jin et al. (2014) [23] reported that the milk protein percentage of Yili horses reached its peak at an energy intake of 106.59 MJ/d, and further increases in energy levels led to a decrease in milk protein percentage. Similar results had been previously shown by Jabbar et al. (2013) [11], who found that milk yield did not increase when the energy level exceeded the required value. The inverse effect of energy on milk production may be related to the direction in which energy is used. Furthermore, a high dietary energy level negatively affects the persistence of lactation. In a previous study, feeding high-energy-level diets to cows easily resulted in spontaneous drying off at the end of lactation compared with cows fed a low-energy-level diet [24]. In this study, the milk production, milk production efficiency, and milk component yield were first increased and then decreased with increasing dietary energy from 11.7 MJ/kg to 13.1 MJ/kg, indicating that the excessively high dietary energy (13.1 MJ/kg) may reduce milk performance.

An increase in DMI is one of the factors contributing to the rise in milk yield. In previous studies, dietary energy concentration had been concluded to affect DMI in the ruminants [25,26]. Zhou et al. (2015) [9] reported that increasing dietary energy density improved DMI of dairy cows. Compared with concentrate, forage has a higher rumen fill value for cows, which indicates that forage consumes lots of space in the digestive tract and reduces feed intake. High energy value and high digestibility of forage allow greater total DMI than the LED of dairy cows [27]. The results of this study indicated that the DMI increased with increasing dietary energy. Although the concentrate-to-forage ratio was similar across the three dietary energy groups, the proportion of alfalfa decreased sequentially from the HED to the LED, while the proportion of millet straw increased accordingly, leading to a sequential reduction in DMI. Zhou et al. (2015) [9] demonstrated that HED enhanced DMI and digestibility of DM, CP, EE, NDF, and ADF, thereby providing adequate energy for lactation and improving milk yield. Cheng et al. (2015) [28] found that HED significantly increased the digestibility of DM and energy in lactating ewes, which is consistent with the current results. It was noted that increasing dietary energy increased DMI, as well as initially increased and then decreased milk performance, which may be attributed to its effects on CP digestibility. Additionally, increasing dietary energy increased the nitrogen metabolic rate and BV, which improved milk protein synthesis efficiency. Generally, higher BUN and MUN levels indicate lower utilization efficiency of dietary nitrogen in animals. A decrease in MUN concentration suggested reduced amino acid catabolism in the mammary gland, thereby improving nitrogen metabolic efficiency [29]. Increasing dietary energy first increased and then decreased the nitrogen metabolic rate, showing an inverse pattern in MUN and serum BUN concentrations. Moreover, MUN was positively correlated with *Lachnospiraceae_AC2044_group* and *unclassified_o__Bacteroidales*, and negatively correlated with *Streptococcus*. It indicated that LED increased *Lachnospiraceae_AC2044_group* and *unclassified_o__Bacteroidales*, and decreased *Streptococcus* to reduce nitrogen metabolism and milk protein synthesis efficiency. This further demonstrates that appropriately increasing dietary energy levels can reduce nitrogen loss, enhance the efficiency of dietary protein conversion into milk protein, and decrease amino acid deamination and urea synthesis. These findings also explain why the MED group exhibited the highest nitrogen metabolic rate and milk protein synthesis efficiency, consequently leading to superior milk performance.

*Clostridiaceae* is highly positively correlated with total dietary energy intake and energy digestibility, but weakly positively correlated with EE digestibility (Bermingham et al., 2017) [30]. In a previous study, we found *Clostridium_sensu_stricto_1* improved the CP, NDF and ADF digestibility, and *Rikenellaceae_RC9_gut_group* decreased the digestibility of CP. *norank_f__norank_o__Bacteroidales* was negatively correlated with DM digestibility [31]. In this study, LED decreased *Clostridiaceae* abundance but increased *Rikenellaceae_RC9_gut_group* and *unclassified_o__Bacteroidales* abundance, which contributed to nutrient digestibility, energy digestibility and metabolism, and caused negative energy balance. Negative energy balance can be evaluated using concentrations of NEFA and BHBA [32]. The reduction in GLU level resulted in lesser INS concentration, which subsequently triggers the fat mobilization process through lipolysis [33]. The present results showed that the serum NEFA and BHBA concentrations decreased with increasing dietary energy, which is consistent with Ferris et al. (2001) [27], reflecting the great demand for GLU and other nutrients by the mammary gland. This enhances body fat mobilization to serve as an energy source for lactation. Furthermore, the concentration of ADPN was increased as body fat decreases, and it promotes glucose uptake. ADPN regulates energy metabolism by promoting fatty acid oxidation and inhibiting the biosynthesis of fat and glucose [34]. In this study, the serum ADPN concentration increased with increasing dietary energy, indicating that adequate dietary energy intake reduced serum NEFA and BHBA concentrations, thereby mitigating negative energy balance. In summary, appropriately increasing dietary energy levels can improve intestinal flora structure, mitigate negative energy balance, and enhance milk yield.

The improved milk production observed in the MED group could also be associated with increased levels of hormones related to lactation. PRL and HYD are primary mediators of mammary cell differentiation and lactogenesis [35,36]. Choi et al. (2004) had also demonstrated that INS promoted the synthesis of both milk and non-milk proteins [37]. A strong positive correlation exists between milk yield and the number of mammary epithelial cells. Lacasse and Petitclerc (2021) [38] reported that elevating serum PRL concentration in dairy cows promoted mammary gland development and enhanced milk production. Sun et al. (2017) [39] found that HYD stimulated milk fat synthesis in bovine mammary epithelial cells. Additionally, serum LEP concentration increased in obese mammals, and LEP inhibited serum-induced proliferation of mammary epithelial cells [40]. This study indicated that increasing dietary energy increased serum concentrations of PRL, INS, and HYD, while decreasing LEP concentrations, which may promote mammary gland cell growth, stimulate the synthesis of milk components, and ultimately result in higher milk yield. Currently, systematic reports on this topic remain scarce, and further investigation is needed.

Therefore, when the dietary DE increased from 11.70 MJ/kg to 13.10 MJ/kg, milk yield, milk component, lactation efficiency, and milk protein synthesis efficiency first increased and then decreased, indicating that the MED group (DE, 12.40 MJ/kg) exhibited better milk performance. Collectively, these findings suggest that a dietary energy level of 12.4 MJ/kg satisfies the optimal nutritional requirements of lactating donkeys, providing a scientific nutritional reference and feasible strategy for the rational feeding practice of donkey production.

### 4.2. Medium-Energy Diet Improved Energy Metabolism and Antioxidative Capability of Lactating Donkeys

In the LED vs. MED comparison, the phenylalanine–tyrosine–tryptophan biosynthesis pathway was significantly enriched, with the level of the differential metabolite L-Phe elevated in the LE group. Phe is both a ketogenic and glucogenic amino acid, and its increase suggests the need to oxidize proteins to meet energy demands, indicating an insufficient energy supply in the LED group. In phenylalanine metabolism, Phe can be converted to hydrocinnamic acid and dihydro-3-coumaric acid, which are further metabolized to succinate for entry into the tricarboxylic acid (TCA) cycle to generate ATP for energy supply. In animals, the primary metabolic pathway for Phe involves its conversion to Tyr via phenylalanine hydroxylase with biopterin as a cofactor [41]. Tyr then exerts biological functions and participates in metabolic regulation [42]. In this study, the LED group produced more L-Phe and L-Tyr through the phenylalanine–tyrosine–tryptophan biosynthesis pathway, providing substrates for phenylalanine and tyrosine metabolism. In the LED group, alterations in phenylalanine metabolism were accompanied by increased levels of L-Phe and L- tyrosine, along with decreased levels of hydrocinnamic acid and dihydro-3-coumaric acid. This indicated enhanced phenylalanine metabolism in the LED group, with less Phe being converted into hydrocinnamic acid and dihydro-3-coumaric acid for direct energy provision, and more Phe being directed toward tyrosine synthesis.

Tyr is also served as a precursor for the synthesis of thyroxine, norepinephrine, and epinephrine [43]. Additionally, tyrosine can be converted to gentisic acid, which is further metabolized to fumarate and participates in the TCA cycle for energy production. Pucci et al. (2000) [44] reported that thyroid hormones promote lipolysis, leading to increased plasma NEFA concentrations. The results of this study showed that, compared with the MED group, the LED group exhibited increased levels of thyroxine, tyramine, and dopaquinone, along with reduced gentisic acid. This suggested that under low dietary energy conditions, less Tyr was converted to gentisic acid for energy provision, while more Tyr was utilized for the synthesis of substances such as thyroxine and dopamine. Elevated levels of thyroid hormones and tyramine facilitated the mobilization of body fat for energy, leading to increased plasma NEFA concentrations. This aligns with the elevated serum NEFA levels with decreasing dietary energy. These results indicate that under negative energy balance, lactating donkeys in the LED group enhanced metabolic pathways such as phenylalanine–tyrosine–tryptophan biosynthesis, phenylalanine metabolism, and tyrosine metabolism to provide energy. However, this compensatory mechanism remained insufficient to offset the energy deficit caused by the low-energy diet.

Coenzyme A (CoA) serves as a crucial cofactor in numerous reversible acetylation reactions involved in carbohydrate, lipid, and amino acid metabolism. Acetyl derivatives such as N-acetylgalactosamine and N-acetylglucosamine exhibit positive correlations with energy balance [45]. Therefore, increased CoA levels were conducive to energy homeostasis in animals. The pantothenate and CoA biosynthesis pathway was significantly enriched in the LED vs. MED, with the differential metabolites pantothenate and L-Val significantly downregulated in the LED. This reduction likely impairs CoA synthesis.

Additionally, biotin content was significantly higher in the MED group than in the LED group, and the biotin metabolism pathway was enriched. These findings suggest that betaine, L-Trp, 3-Methyl-L-His, pantothenate, L-Val, and biotin are key differential metabolites associated with negative energy balance and reduced milk production induced by low-energy diets in lactating donkeys. Furthermore, the ascorbate and aldarate metabolism pathway and glutathione metabolism pathway were enriched in the same comparison, with the differential metabolites D-glucuronic acid and glutathione downregulated in the LED group. This reduction likely compromises the antioxidant capacity of the LED group. Simultaneously, L-His, a differential metabolite in histidine metabolism, was reduced in the LED group. Histidine exerts anti-inflammatory effects by inhibiting NF-κB activation and downregulating pro-inflammatory cytokine production [46], as well as by activating PPARγ to enhance adiponectin secretion. Additionally, studies indicate that histidine serves as a structural and functional unit of hemoglobin and acts as a precursor for synthesizing antioxidant dipeptides [47]. Consequently, the phenylalanine-tyrosine-tryptophan biosynthesis, tryptophan metabolism, and histidine metabolism pathways are critical metabolic routes. L-Trp, L-His, D-glucuronic acid, and glutathione were identified as key differential metabolites contributing to diminished antioxidant capacity and milk production performance in lactating donkeys under LED conditions.

In the comparison between the LED and MED groups, integrated proteomic-metabolomic analysis revealed enrichment of the thyroid hormone synthesis, tyrosine metabolism, and glutathione metabolism pathways. These pathways can enhance energy metabolism and antioxidant function, thereby improving the milk production performance of lactating donkeys. ALB, TPO, and cAMP promote the synthesis of thyroxine, and their elevated levels in the MED group indicate that the MED enhances energy metabolism in lactating donkeys through the thyroid hormone synthesis and tyrosine metabolism pathways, providing more energy for lactation. Simultaneously, the activities of GSH-Px, SOD, and T-AOC increased with increasing dietary energy, which suggests that the medium-energy diets improved the antioxidant capacity of lactating donkeys through the glutathione metabolism pathway, thereby contributing to enhanced milk production performance.

According to KEGG pathway analysis, in histidine metabolism, amine oxidase mediates the conversion of histidine to aspartate, which subsequently enters the tricarboxylic acid cycle through alanine, aspartate, and glutamate metabolism to generate energy. Amine oxidase degraded amino propanone, a metabolite of glycine and threonine. Aminopropanone promoted the formation of methylglyoxal, a compound with cytotoxic and genotoxic properties, and induced cellular oxidative damage [48]. Reduced amine oxidase in the LED group may lead to aminopropanone accumulation, potentially causing damage to mammary gland cells. In tryptophan metabolism, decreased L-Trp levels in the LED group may reduce the production of N-acetylserotonin, which has been reported to promote prolactin secretion [49] and facilitate the release of thyroxine and norepinephrine [50]. These changes may further influence lactation performance and metabolic regulation. While this study investigated how LED, compared with MED, regulates production performance, negative energy balance, and antioxidant function in lactating donkeys, it did not explore the mechanisms by which HED also reduces these traits. Therefore, the regulatory mechanism of dietary energy level on lactation performance and antioxidant function in lactating donkeys cannot be fully elucidated, and further investigation is warranted.

## 5. Conclusions

Increasing dietary energy from 11.7 MJ/kg to 13.10 MJ/kg resulted in a quadratic response (increasing and decreasing) in lactation performance, nutrient digestion, and antioxidative capacity, with the highest values observed at 12.4 MJ/kg. Changes in the composition of the rectal microbiota, metabolites and proteins may lead to this condition. Thyroid hormone synthesis, tyrosine metabolism, and glutathione metabolism pathways are critical metabolic routes; these pathways can enhance energy metabolism and antioxidant function, thereby improving the milk production performance of lactating donkeys. In summary, these findings suggest that a dietary energy level of 12.4 MJ/kg satisfies the optimal nutritional requirements of lactating donkeys, providing a scientific nutritional reference and feasible strategy for the rational feeding practice of donkey production.

## Figures and Tables

**Table 1:** Experimental diet composition and nutritional level (air-dry basis).

Items	HED ^3^	MED ^3^	LED ^3^
Feed Ingredients (%)			
Millet straw	26.91	38.79	54.76
Alfalfa	29.99	17.94	4.48
Corn silage	13.12	13.07	10.44
Corn	18.18	15.60	11.16
Soybean meal	0.00	7.19	5.35
Corn gluten meal	0.00	1.86	5.72
Corn germ meal	0.16	1.55	3.09
DDGS	0.31	0.46	0.00
Bran	0.30	1.49	2.98
Extruded full-fat soybean	9.00	0.00	0.00
Sodium chloride	0.40	0.40	0.40
Limestone powder	0.36	0.36	0.36
CaHPO_4_	0.72	0.72	0.72
Premix ^1^	0.55	0.55	0.55
Total	100.00	100.00	100.00
Nutrient level ^2^ (%)			
DE (MJ/kg)	13.10	12.40	11.70
CP	14.01	14.01	14.02
EE	4.46	2.82	2.96
NDF	49.75	52.48	55.53
ADF	30.30	31.15	31.89
Ca	1.16	1.11	1.08
P	0.36	0.37	0.36

^1^ Provided per kg of premix: VA 6,000,000 IU, VD 1,250,000 IU, VE 15,000 IU, Fe 20 g, Cu 8.0 g, Zn 60.0 g, Mn 60 g, I 360 mg, Se 300 mg, and Co 500 mg. ^2^ Digestible energy is calculated based on the ingredients of the diet and their digestible energy content, not based on the actual dry matter intake. ^3^ HED = high-energy diet, MED = medium-energy diet, and LED = low-energy diet.

**Table 2:** Effect of dietary energy level on DMI and milk performance in lactating donkeys.

Item ^1^	Dietary Energy ^2^			SEM ^3^	Contrast ^4^	
	HED	MED	LED		Linear	Quadratic
DMI, kg/d	8.02	7.20	6.80	0.29	<0.001	<0.001
Milk production						
Milk Yield, kg/d	0.86	0.93	0.75	0.024	0.001	0.002
EMY, kg/d	3.43	3.71	2.99	0.096	0.001	0.003
ECM, kg/d	1.65	1.88	1.47	0.052	<0.001	<0.001
SCM, kg/d	1.75	2.03	1.49	0.086	0.030	0.049
Milk production efficiency, yield/intake						
EMY/DMI	0.43	0.53	0.46	0.010	<0.001	<0.001
ECM/DMI	0.21	0.26	0.22	0.005	0.001	<0.001
SCM/DMI	0.22	0.28	0.22	0.010	0.003	0.003
Milk protein synthesis efficiency	0.78	0.92	0.72	0.020	<0.001	<0.001
Milk component						
Fat, %	0.29	0.32	0.28	0.010	0.102	0.108
Protein, %	1.66	1.74	1.70	0.015	0.019	0.023
Lactose, %	6.57	6.52	6.50	0.020	0.005	0.026
SNF, %	8.82	8.85	8.81	0.015	0.184	0.183
TS, %	9.11	9.18	9.13	0.022	0.032	0.031
MUN, mg/dL	30.14	28.96	33.79	0.810	0.054	0.002
Milk component yield						
Fat, g/d	8.89	12.20	8.00	0.47	0.010	0.014
Protein, g/d	55.33	65.86	49.30	1.85	0.002	0.003
Lactose, g/d	214.41	244.53	183.30	5.95	0.011	0.021
SNF, g/d	284.76	328.27	243.47	13.76	0.015	0.022
TS, g/d	294.62	340.26	250.80	14.10	0.014	0.021

^1^ DMI = dry matter intake, EMY = estimated milk yield, ECM= energy-corrected milk, SCM = solids-corrected milk. SNF = solids not fat, TS = total solids, MUN = milk urea-N. ^2^ HED = high-energy diet, MED = medium-energy diet, LED = low-energy diet; ^3^ SEM = standard error of the mean; ^4^ Contrasts correspond to the linear and quadratic effects of increasing dietary energy; Significance was declared at *p* < 0.05, and trends were noted at 0.05 < *p* ≤ 0.10. No significant interaction effects between dietary energy level and lactation week were observed for any variables in this table (*p* > 0.05).

**Table 3:** Effect of dietary energy level on nutrient digestibility, energy metabolism and nitrogen metabolism in lactating donkeys (%).

Item ^1^	Dietary Energy ^2^			SEM ^3^	Contrast ^4^	
	HED ^2^	MED	LED		Linear	Quadratic
DM	73.17	72.84	70.47	0.38	<0.001	0.057
CP	79.23	82.62	81.41	1.01	0.034	0.012
EE	59.56	51.77	46.45	1.36	<0.001	0.642
NDF	58.61	57.24	50.71	1.52	0.003	0.211
ADF	52.40	52.78	44.54	1.50	<0.001	0.013
Ca	58.58	57.58	66.61	1.63	0.001	0.009
P	46.60	52.44	57.72	2.34	0.002	0.926
energy digestibility	64.85	66.56	56.98	1.24	<0.001	0.001
energy metabolism	62.67	62.34	53.81	1.51	<0.001	0.001
nitrogen metabolism	71.80	73.15	69.04	0.96	0.058	0.032
BV	87.37	87.96	85.24	0.63	0.027	0.045

^1^ DM = dry matter, CP = crude protein, EE = ether extract, NDF = neutral detergent fiber, ADF = acid detergent fiber, Ca = calcium, P = phosphorous, BV= Biological value of proteins. ^2^ HED = high-energy diet, MED = medium-energy diet, LED = low-energy diet. ^3^ SEM = standard error of the mean. ^4^ Contrasts correspond to the linear and quadratic effects of increasing dietary energy. Significance was declared at *p* < 0.05, and trends were noted at 0.05 < *p* ≤ 0.10.

**Table 4:** Effect of dietary energy level on serum biochemical indicatorsin lactating donkeys.

Item ^1^	Dietary Energy ^2^			SEM ^3^	Contrast ^4^	
	HED	MED	LED		Linear	Quadratic
TP, g/L	59.38	60.75	60.75	1.07	0.372	0.604
ALB, g/L	25.88	26.13	25.38	0.43	0.424	0.357
BUN, mmol/L	8.42	8.09	8.96	0.18	0.030	0.001
AST, U/L	5.00	4.20	4.17	0.26	0.036	0.248
ALP, U/L	181.86	158.50	123.86	10.57	0.002	0.704
ALT, U/L	378.33	315.50	335.00	14.91	0.064	0.046
TG, mmol/L	0.60	0.57	0.51	0.02	0.001	0.563
TBIL, μmol/L	7.38	7.50	7.50	0.19	0.641	0.788
CHO, mmol/L	1.97	1.66	1.56	0.08	0.002	0.305
HDL-C, mmol/L	0.67	0.81	0.64	0.03	0.560	0.002
LDL-C, mmol/L	0.18	0.13	0.14	0.01	0.045	0.051
GLU, mmol/L	3.74	3.51	3.43	0.05	0.005	0.336
BHBA, mmol/L	0.29	0.42	0.65	0.08	0.016	0.598
NEFA, mmol/L	191.44	297.00	402.67	35.35	0.022	0.871

^1^ TP = total protein, ALB = albumin, BUN= blood urea nitrogen, AST = aspartate aminotransferase, ALP = alkaline phosphatase, ALT = alanine aminotransferase, TG = triglyceride, TBIL = total bilirubin, CHO = total cholesterol, HDL-C = high-density lipoprotein cholesterol, LDL-C = low-density lipoprotein cholesterol, GLU = glucose, BHBA = β-hydroxybutyric acid, NEFA = non-esterified fatty acids. ^2^ HED = high-energy diet, MED = medium-energy diet, LED = low-energy diet. ^3^ SEM = standard error of the mean. ^4^ Contrasts correspond to the linear and quadratic effects of increasing dietary energy. Significance was declared at *p* < 0.05, and trends were noted at 0.05 < *p* ≤ 0.10.

**Table 5:** Effect of dietary energy level on serum hormones and antioxidant indicators of lactating donkeys.

Item ^1^	Dietary Energy ^2^			SEM ^3^	Contrast ^4^	
	HED	MED	LED		Linear	Quadratic
Hormone						
PRL, ng/mL	126.39	161.47	115.61	10.47	0.504	0.009
INS, mIU/L	15.58	19.36	11.41	1.60	0.118	0.026
LEP, ng/mL	3.11	2.40	2.00	0.15	0.562	0.001
GC, pg/mL	68.88	75.2	77.95	3.33	0.067	0.678
ADPN, ug/mL	5.68	4.79	8.61	0.65	0.004	0.007
HYD, ng/mL	1.74	2.30	1.77	0.16	0.899	0.012
Antioxidant						
GPx (U/mL)	281.56	298.74	289.01	2.963	0.090	0.001
TrxR (U/mL)	49.17	53.31	50.74	1.612	0.500	0.104
SOD (U/mL)	71.61	85.60	68.75	3.845	0.605	0.004
T-AOC (mM)	0.30	0.35	0.34	0.011	0.018	0.020
MDA/(nmol/mL)	1.93	1.67	1.58	0.079	0.011	0.311

^1^ PRL = prolactin, INS = insulin, LEP = leptin, GC = glucagon, ADPN = adiponectin, HYD = hydrocortisone; GPx = glutathione peroxidase, TrxR = thioredoxin reductase CAT = catalase; SOD = superoxide dismutase; T-AOC = total antioxidant capacity; MDA = malondialdehyde; ^2^ HED = high-energy diet, MED = medium-energy diet, LED = low-energy diet. ^3^ SEM = standard error of the mean. ^4^ Contrasts correspond to the linear and quadratic effects of increasing dietary energy. Significance was declared at *p* < 0.05, and trends were noted at 0.05 < *p* ≤ 0.10.

**Table 6:** The alpha diversity indexes of bacteria in rectal digesta.

Items ^1^	HED ^2^	MED	LED	SEM ^3^	*p*-Value
Sobs	1258.33 ^b^	1420.17 ^a^	1402.00 ^a^	27.28	0.001
ACE	1547.51 ^b^	1677.04 ^a^	1665.96 ^a^	27.37	0.008
Chao	1558.41 ^b^	1696.85 ^a^	1684.89 ^a^	29.18	0.008
Shannon	4.48 ^b^	5.79 ^a^	5.82 ^a^	0.24	0.002
Simpson	0.15 ^a^	0.01 ^b^	0.01 ^b^	0.03	0.003
Coverage	0.9895	0.9897	0.9897	0.0003	0.842

^a,b^ Different superscript letters within the same row indicate a significant difference between experimental groups (*p* < 0.05). ^1^ Sobs = observed species richness; ACE = abundance-based coverage estimator; Chao = Chao1 richness estimator; Shannon = Shannon–Wiener diversity index; Simpson = Simpson diversity index; Coverage = sequencing coverage. ^2^ HED = high-energy diet, MED = medium-energy diet, LED = low-energy diet. ^3^ SEM = standard error of the mean.

**Table 7:** Effect of dietary energy level on serum metabolites in lactating donkeys.

KEGG Metabolic Pathways	Total	Hits	Impact	*p*	Upregulated	Downregulated
Phenylalanine metabolism	51	3	0.099	0.003	L-Phenylalanine	Hydrocinnamic acid Dihydro-3-coumaric acid
Phenylalanine, tyrosine and tryptophan biosynthesis	34	2	0.002	0.016	L-Phenylalanine	Fructose 1-phosphate
Tyrosine metabolism	60	2	0	0.044	Phenol, Tyramine	
Nicotinate and nicotinamide metabolism	50	1	0.064	0.230		Niacinamide

**Table 8:** The association analysis of serum key differential metabolites with NEFA and BHBA.

Metabolite	NEFA		BHBA	
	R	*p*	R	*p*
Hydrocinnamic acid	−0.597	0.002	−0.494	0.014
L-Phenylalanine	0.416	0.043	0.376	0.070
Dihydro-3-coumaric acid	−0.190	0.372	−0.475	0.019

**Table 9:** Effect of dietary energy level on milk metabolites in lactating donkeys (LED vs. MED).

KEGG Metabolic Pathways	Total	Hits ^1^	Impact	*p*-Values ^2^	Upregulated	Downregulated
Tryptophan metabolism	54	4	0.232	0.001		Indoleacetaldehyde 2-Aminobenzoic acid N-Acetylserotonin L-Tryptophan
Phenylalanine, tyrosine and tryptophan biosynthesis	34	3	0.063	0.002		2-Aminobenzoic acid 4-Hydroxyphenylpyruvic acid L-Tryptophan
Pantothenate and CoA biosynthesis	25	2	0.152	0.017		Pantothenic Acid, L-Valine
Arginine and proline metabolism	72	3	0.112	0.018		L-Proline, 2-Oxoarginine 4-Guanidinobutanoic acid
Histidine metabolism	33	2	0.154	0.029		L-Histidine 3-Methyl-L-histidine
Glycine, serine and threonine metabolism	47	2	0.024	0.052	Betaine	L-Tryptophan
Glutathione metabolism	38	1	0.279	0.241		Glutathione
Biotin metabolism	23	1	0.081	0.165		Biotin
Ascorbate and aldarate metabolism	43	3	0.011	0.261		D-Glucarate

^1^ Hits represent the number of KEGG compound IDs annotated to pathways in this metabolism; ^2^ The *p*-value is uncorrected, and *p*-values less than 0.05 are considered enrichment terms.

**Table 10:** The association analysis of milk key differential metabolites with NEFA and BHBA.

Metabolite	NEFA		BHBA	
	R	*p*	R	*p*
L-Tryptophan	−0.064	0.765	−0.391	0.059
D-Glucarate	−0.304	0.148	−0.364	0.080
Biotin	−0.255	0.229	−0.399	0.053
Niacinamide	−0.295	0.162	−0.415	0.044
L-Tyrosine	0.350	0.093	0.009	0.964
Glutathione	−0.403	0.051	−0.436	0.033
3-Methyl-L-histidine	−0.459	0.024	−0.389	0.060
Betaine	0.188	0.377	0.096	0.653
Ascorbic acid	0.322	0.124	0.143	0.503
UDP-D-galactose	−0.143	0.504	−0.174	0.414
L-Histidine	−0.121	0.571	−0.218	0.306

**Table 11:** Enrichment of partial KEGG pathways of LED vs. MED.

KEGG Pathways Name	Hits ^1^	*p*_Value ^2^	Upregulated	Downregulated
Thyroid hormone synthesis	8	0.005	SERPIN domain-containing protein (A0A3Q2HM80)	Glutathione peroxidase (A0A5F5PST7) G protein subunit alpha q (A0A3Q2IAY3) Afamin (A0A3Q2ID60) Serpin family A member 6 (F7DRS2) Serpin family A member 3 (F6ZLR1) Lactoperoxidase (F7C3Z3) G protein subunit alpha 12 (A0A3Q2H8B8)
Glutathione metabolism	3	0.024		Glutathione S-transferase (A0A5F5PSQ4) Glutathione peroxidase (A0A5F5PST7) Glutathione transferase (M9ZUR8)
Galactose metabolism	4	0.069	Glucosidase II alpha subunit (F7C4H8) Beta-galactosidase (A0A3Q2IDA4) Glucosidase alpha, acid (F6W5W1) Alpha-galactosidase (A0A3Q2HXM5)	
PI3K-Akt signaling pathway	1	0.980		Rac family small GTPase 1 (A0A3Q2HKU0)

^1^ Hits represent the number of KEGG compound IDs annotated to pathways in this metabolism; ^2^ The *p*-value is uncorrected, and *p*-values less than 0.05 are considered enrichment terms.

**Table 12:** The partial KEGG pathways involved by differential proteins and differential metabolites in LED vs. MED.

Pathway Name	Upregulated Protein	Downregulated Protein	*p*_Value ^1^	Upregulated Metabolite	Downregulated Metabolite	*p*_Value ^1^
Thyroid hormone synthesis	A0A3Q2HM80	F7C3Z3, F7DRS2 A0A3Q2ID60 A0A3Q2H8B8 A0A5F5PST7 A0A3Q2IAY3 F6ZLR1	0.005	-	Glutathione, CAMP	0.016
Tyrosine metabolism	-	F7C3Z3, F7DLT3	0.149	-	4-Hydroxyphenylpyruvic acid	0.521
Glutathione metabolism	-	A0A5F5PSQ4 A0A5F5PST7 M9ZUR8	0.024	-	Glutathione	0.300
Glycine, serine and threonine metabolism	-	F7DLT3	0.464	Betaine	L-Tryptophan	0.078
Vitamin digestion and absorption	-	A0A3Q2I5H0 F6Z2L5	0.238	-	Pantothenic Acid, Biotin, Niacinamide	0.005
Protein digestion and absorption	-	A0A5F5PQX9 F6ZSR4	0.576	-	L-Valine, L-Histidine L-Tryptophan, L-Proline L-Isoleucine	0.0001
Histidine metabolism		F7DLT3	0.312		L-Histidine 3-Methyl-L-histidine	0.070
Tryptophan metabolism	-	F7DLT3	0.464	Indoleacetaldehyde	Tryptophanol N-Acetylserotonin 2-Aminobenzoic acid L-Tryptophan	0.0008

^1^ The *p*-value is uncorrected, and *p*-values less than 0.05 are considered enrichment terms.

## Data Availability

The raw sequencing data are available at the National Center for Biotechnology Information database (Bio Project ID: PRJNA1430129).
